# Editorial: New Trends on Genome and Transcriptome Characterizations

**DOI:** 10.3389/fgene.2018.00322

**Published:** 2018-08-20

**Authors:** Rosalba Giugno, Vincenzo Manca

**Affiliations:** Department of Computer Science, University of Verona, Verona, Italy

**Keywords:** algorithms, data analysis, computational biology, bioinformatics, analysis methods

Worldwide, National Health Systems are investing to fit the requirements of “precision medicine.” This term refers to the prevention and treatment of diseases that take into account the individual characteristics of patients, from their genetic variability to their different life style. However, the aims of precision medicine can only be fully realized when the internal mechanisms of diseases are understood and a deep knowledge of their individual variability is reached. Such a high level of comprehension can not only allow physicians to maximize the benefit against dangerous side effects, but can also promote the discovery of new treatments and prevention procedures. The same attitude, which in medicine can be synthetized as a shift from pathologies to patients, can be applied in the field of agriculture, when general principles are adapted and regulated according to the specific environments and local situations where cultures are realized.

These objectives require financial and intellectual resources to collect large amounts of genome and transcriptome data that are specifically related to the phenomena of interest in the different fields of applications (diseases in many contexts of related pathologies, or agricultural settings at the production level). However, a second methodological aspect is the powerful combination of information theoretical concepts with specific algorithmic and computational tools. This informational perspective is intended to extract deep biological meaning that often escape from simple statistical analyses of macroscopic phenomena. In fact, long range correlations or deep mathematical regularities, surprisingly enough, seem to relate with biological structures and functions that are encoded in a multilevel organization of genomes and of their expression. The search for new information-based categories will provide novel interpretation of classical biological concepts using this new informational approach.

The results of this methodological innovation are going to have a wide range of applications in the public health and in many economic sectors that contribute positively to human lifestyle and to progress of countries. In particular, in the agri-food sector, the study of the variability of genomes and transcriptomes implies the improvement of productivity and quality of products. In this field the combination of plant biotechnology with bioinformatics, in comparison with the traditional techniques of phenotypic analysis of plants, will provide a remarkable increase of speed and efficiency in the selection of progenies with superior characteristics.

This Research Topic collected contributions from computer scientists, bioinformaticians, and geneticists who designed or applied unconventional methods to understand pathology in medical and agricultural contexts. The issue comprises 12 articles, with 9 original research articles and 3 reviews.

Mosca et al. present a network smoothing method to predict gene modules involved in Alzheimer disease. The approach prioritizes the role of genes in the disease by the grade of their smoothing, which reflect the interaction topology of those genes with known genes in the AD. It is interesting to notice that, while the paper was under review, 3 of the several genes predicted in this work were, independently, added to the genes-association database (SFARI) of The Simons Foundation Autism Research Initiative.

Pedersen et al. present an integrative approach to combine information from tissue specific protein interaction networks with genome-wide gene association in Type 2 diabetes (T2D). They first constructed pancreatic and beta-cell protein complexes to be used as reference model for scaffold procedures, and then, by means of them, identified a set of 24 islet protein complexes probably disregulated or disfunctioning in T2D. Human specific tissues are investigated in Mayne et al. to assess the variegation of diseases depending on sex. By making use of meta-analysis approach, authors demonstrate that, among other results, even cortical regions may influence sexually dimorphic traits. Moreover, a large percentage of genes whose expression was sex-biased had androgen or estrogen hormone response elements.

Establishing the exceptional method for calculating deregulated genes has attracted many research efforts and we expect to see other attempts, in particular in the emerging scenario of single-cell RNA sequencing. Dal Molin et al. compare four different tools dedicated to differential expression of single cell RNA sequencing and extended two methods, commonly used for single cell data, for the analysis of bulk RNA sequencing data. The results on real and synthetic datasets (which imitate unimodal and bimodal distributions) reveal the limitations of each tool, by showing that no tool outperforms the others. Bulk RNA sequencing data related to ovarian follicle are deeply analyzed by Battaglia et al., a team devoted to this subject, aimed at characterizing non-coding RNAs to improve medical practice in infertility disorders, concerning with diagnosis, treatment, and discovery of biomarkers, for oocyte quality, in Assisted Reproductive Treatment. Computational methods in agriculture, combining bulk RNA sequencing and advanced bioinformatics methods, are presented by Wang et al. to detect new small RNAs and other interfering RNAs having a role in the tomato ethylene signaling pathway and fruit ripening.

Chen et al. investigate the genome duplication in rosids, whereas Cao et al. provide to assess the evolution of WUSCHEL-related homeobox transcription factors in rosaceae. Alagarasan et al. characterize the ZIP gene family in Setaria italic. Three reviews complete the issue. Chiara and Pavesi present the best practices for read pre-processing in the identification of human variome, i.e., casual mutations of haplotypes, in order to overcome the lost quality due to the high variability of data (gene panels, exomes, or whole genomes).

Vandin presents computational methods, mostly involving network analysis, for characterizing inter-tumor heterogeneity coming from pathways commonly mutated across different patients, and intra-tumor heterogeneity coming from bulk or single cell sequencing data. Finally, Alaimo et al. review computational techniques in agriculture area for grapevine meta-omics analysis. Authors discussed also the current knowledge of microbiome in plants, its differences varying according to the parts of plants, and its role to cause or protect from diseases.

## Author contributions

RG and VM handled manuscripts and edited the Research Topic.

### Conflict of interest statement

The authors declare that the research was conducted in the absence of any commercial or financial relationships that could be construed as a potential conflict of interest.

